# The Oral–Gut–Brain Axis: The Influence of Microbes as a Link of Periodontitis With Ischemic Stroke

**DOI:** 10.1111/cns.70152

**Published:** 2024-12-15

**Authors:** Yi Zhong, Xianhui Kang, Xiaofeng Bai, Bei Pu, Daniel Smerin, Liang Zhao, Xiaoxing Xiong

**Affiliations:** ^1^ Department of Neurosurgery Renmin Hospital of Wuhan University Wuhan China; ^2^ Department of Anesthesiology, Union Hospital, Tongji Medical College Huazhong University of Science and Technology Wuhan China; ^3^ Department of Anesthesiology, the First Affiliated Hospital, College of Medicine Zhejiang University Hangzhou Zhejiang China; ^4^ Department of Oral and Maxillofacial Surgery, Stomatology Hospital Zhejiang University School of Medicine Zhejiang China; ^5^ Department of Neurosurgery University of Texas Health Science Center at San Antonio San Antonio Texas USA; ^6^ Department of Gastroenterology Renmin Hospital of Wuhan University Wuhan China

**Keywords:** ischemic stroke, microbiota, oral–gut–brain axis, periodontitis

## Abstract

Periodontitis, a non‐communicable chronic inflammation disease resulting from dysbiosis of the oral microbiota, has been demonstrated to have a positive association with the risk of ischemic stroke (IS). The major periodontal pathogens contribute to the progression of stroke‐related risk factors such as obesity, diabetes, atherosclerosis, and hypertension. Transcriptional changes in periodontitis pathogens have been detected in oral samples from stroke patients, suggesting a new conceptual framework involving microorganisms. The bidirectional regulation between the gut and the central nervous system (CNS) is mediated by interactions between intestinal microflora and brain cells. The connection between the oral cavity and gut through microbiota indicates that the oral microbial community may play a role in mediating complex communication between the oral cavity and the CNS; however, underlying mechanisms have yet to be fully understood. In this review, we present an overview of key concepts and potential mechanisms of interaction between the oral–gut–brain axis based on previous research, focusing on how the oral microbiome (especially the periodontal pathogens) impacts IS and its risk factors, as well as the mediating role of immune system homeostasis, and providing potential preventive and therapeutic approaches.

## Graphical abstract

The underlying mechanism of oral–gut–brain axis between periodontitis and ischemic stroke could occur through the following pathways: (1) direct transfer of bacteria and their products via nervous or blood; (2) gut dysbiosis due to imbalanced oral microbiota; and (3) activation of inflammatory and immune responses activation by the secretion of inflammatory mediators.

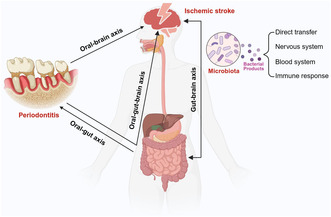

**Abbreviations**
AD: Alzheimer's disease
AgP: aggressive periodontitis
AMX: amoxicillin
ApoE: apolipoprotein E
CNS: central nervous system
CP: chronic periodontitis
CRP: C‐reactive protein
DCs: dendritic cells
EMD: enamel matrix derivatives
EPS: exopolysaccharides
F/B: Firmicutes/Bacteroidetes
GF: germ‐free
HP: hazard ratio
HPA: hypothalamic–pituitary–adrenal
IBD: inflammatory bowel disease
IL: interleukin
IS: ischemic stroke
LPS: lipopolysaccharides
MET: metronidazole
NSAIDs: non‐steroidal anti‐inflammatory drugs
OMVs: outer membrane vesicles
PPI: proton pump inhibitors
rhPDGF‐BB: recombinant human platelet‐derived growth factor BB
SAP: stroke‐associated pneumonia
SRP: scaling and root planing
T2DM: type 2 diabetes
TLRs: toll‐like receptors
TMAO: trimetlylamine oxide
TNF: tumor necrosis factor
Treg: T regulatory

## 1 Introduction

The gut microbiome and the oral microbiome are two of the most ecologically rich and taxonomically diverse microbiomes in the human body [1]. Accumulating evidence supports bidirectional communication between the gut and the brain mediated by gut microbes, which is defined as the gut–brain–microbiome axis, and disorders of which are associated with various CNS diseases [2, 3]. A potential oral–gut–brain axis has been proposed. Oral bacteria migrate to the digestive tract to form ectopic colonies, which may lead to intestinal imbalance [4]. Oral bacteria and their products may also directly affect the brain through the nervous system or blood circulation [5]. Like within the intestinal flora, maintaining homeostasis within the oral microecosystem is crucial for host health. Understanding microbiome factors and deciphering host‐bacterial interactions are essential for furthering our understanding of disease etiology and facilitating biomarker development for prevention and treatment.

An increasing number of studies demonstrates the bidirectional relationship between oral diseases and cerebrovascular diseases [5]. Periodontitis, one of the most prevalent chronic inflammatory diseases of the oral cavity, is triggered by pathogenic bacteria residing in subgingival sulcus. The dysbiosis of oral microorganisms not only leads to periodontal tissue destruction but also contributes to systemic inflammation [6]. Numerous clinical and basic investigations have established a connection between periodontitis and ischemic stroke (IS), revealing that individuals with periodontitis face a significantly elevated risk of IS event [7, 8, 9]. Moreover, stroke survivors with periodontitis exhibit poorer prognoses and are more susceptible to recurrent vascular events [10]. However, the underlying mechanism linking these conditions remains unclear. This review aims to comprehensively elucidate the connection between IS and periodontitis while exploring the potential impact of the oral–gut–brain axis—an essential aspect for early intervention and subsequent treatment strategies for IS.

## 2 Oral Microbiota

The human body harbors diverse microbial communities, and statistical data indicate that the cell‐to‐microbial ratio in different anatomical sites is approximately 1:10 [11]. There are four primary microbial communities: the oral cavity, gastrointestinal tract, skin, and vagina. The oral cavity represents the second largest reservoir of microorganisms following the gut [12]. The oral microbiome primarily inhabits the dental surfaces, tongue, gingival sulcus, and tonsils; encompassing a diverse array of bacteria, viruses, fungi, protozoa, and archaea [13]. According to the 16S rDNA profiling of the healthy human oral cavity, a total of 700 bacterial species belonging to 185 genera and 12 phyla have been identified [14]. The *Firmicutes*, *Actinobacteria, Proteobacteria*, *Fusobacteria*, *Bacteroidetes*, and *Spirochaetes* constitute 80%–95% of the total oralbacterial population. *Actinomyces*, *Prevotella*, *Streptococcus*, *Fusobacterium*, and *Veillonella* represent the predominant strains inhabiting the oral cavity [13, 15, 16]. Most oral microbiota coexist and thrive within oral biofilms, with the exception being saliva [17]. Biofilms are defined as bacterial aggregates adhering to an inert or biological surface; composed of proteins, lipids, polysaccharides, and extracellular DNA, and enveloped by an extracellular polymeric matrix. Also, biofilms serve as microenvironments capable of regulating pH, oxygen levels, and redox status in the oral cavity while enhancing nutrient availability for bacteria and safeguarding them against environmental stressors, thus establishing a delicate equilibrium between pathogens and symbiotic microorganisms [18].

In addition to bacteria, the oral cavity is also inhabited by indigenous viruses, fungi, and yeasts; however, limited information is available regarding these oral biomes compared to the bacterial microbiota. The most prevalent viral families found in healthy individuals include *Anelloviridae*, *Papillomaviridae*, and *Herpesviriade* [19]. It is estimated that over 100 fungal species colonize the oral cavity, and only under specific conditions (particularly immunocompromised individuals or drug abuse) can they manifest as opportunistic pathogens [20, 21].

## 3 Oral Microbiota and Periodontitis

As consistently demonstrated by numerous studies, the oral microbiota plays a pivotal role in the pathogenesis of oral diseases. Disruption of the homeostasis in oral flora can result in an imbalance between pathogenic and saprophytic microorganisms within the oral cavity, potentially causing oral diseases such as periodontitis [22]. The prevalence range of periodontitis spans from 20% to 85% among the general population [23], representing a polymicrobial infection affecting the periodontal tissues due to the presence of plaque biofilm on both teeth and gingiva. As an arsenal for bacterial pathogenesis, plaque generates antigens that invade the deep gums and impair the host immune defense system, thus initiating an inflammatory response. Inflammatory tissue breakdown products such as degraded collagen and heme‐containing compounds are released into the gingival crevicular fluid, serving as nutrients to fuel the selective proliferation of pathogens and further exacerbate microbiota imbalance, leading to irreversible destruction of tooth‐supporting tissues, characterized by clinical manifestations such as attachment loss, formation of periodontal pockets, and resorption of alveolar bone [24, 25, 26]. If left untreated, these conditions can progressively result in tooth mobility or even tooth loss [27, 28]. As periodontitis progresses, the progressive destruction of gingiva, bone, and ligaments occurs and ultimately results in systemic inflammation. In 1998, Socransky et al. [29] proposed the “red complex” represented by *Porphyromonas gingivalis* , *Tannerella forsythia* , and *Treponema denticola* as the pathogenic bacteria responsible for periodontitis, which exhibit a close association with the clinical manifestation and symptoms of periodontitis and are strongly correlated with disease severity. Among them, *P. gingivalis* plays a pivotal role as a keystone pathogen in oral microbiota imbalance and the development of periodontitis [30]. It was detected in 85.75% subgingival plaque of chronic periodontitis (CP) patients [31]. Even at low abundance (< 0.01% of the total bacterial count), *P. gingivalis* can disrupt host immune response and disturb the symbiotic relationship among local oral bacteria [32]. The pathogenicity of *P. gingivalis* heavily relies on its expressed or released virulence factors including lipopolysaccharides (LPS), fimbriae, capsules, and gingipains. LPS, also referred to as endotoxin, constitutes a prominent constituent of the cell walls in Gram‐negative bacteria, and the core component of which, Lipid A, triggers inflammatory responses in periodontal tissue by binding to specific Toll‐like receptors (TLRs) on the surface of host cells and activating downstream signaling pathways [33]. Fimbriae facilitate biofilm formation, bacterial motility, adhesion to host cells, and aggregation with other organisms. Capsules aid *P. gingivalis* in evading host immune defenses while enhancing invasion along with fimbriae [34]. Gingipains play a role in various aspects of peridontal gingivalis infection such as nutrient acquisition for bacterial growth, tissue destruction promoting invasion, and degradation of host immune factors [35]. Collectively, these virulence factors create an optimal environment for pathogen persistence and periodontal disease development. Furthermore, correlations between periodontitis and other pathogens have been established in recent years, including *Filifactor alocis* , *Synergistetes*, *Peptostreptococcaceae*, and *Aggregatibacter actinomycetemcomitans* which are associated with aggressive forms of periodontitis [36]. A direct relationship between periodontal inflammation and reduced levels of *Lactobacilli* was also observed. Specifically, patients with CP exhibited lower abundances of bacteria such as *L. fermentum* and *L. gasseri* compared to healthy individuals [37, 38]. The latest research has indicated that the dysbiosis of oral microbiota can also impact individual microbial communities, thereby establishing periodontitis as a potential risk factor for various systemic diseases including cardio‐cerebrovascular diseases, metabolic diseases, and autoimmune diseases [6, 39].

## 4 The Cross Link Between Oral Microbiota, Periodontitis, and Ischemic Stroke

### 4.1 Periodontitis and Ischemic Stroke

IS is a sudden onset of localized neurological dysfunction caused by abnormal occlusion of cerebral blood flow and is a leading cause of death and disability globally. Available data have demonstrated a positive association between periodontitis and stroke. A meta‐analysis comprising five case–control studies and three cohort studies revealed that the hazard ratio (HR) for IS in individuals with periodontitis was 2.88 (95% confidence interval [CI] 1.53–5.41) [7]. Furthermore, pooled relative risk estimates from cohort studies indicated an overall relative risk of 2.52 (1.77–3.58) and an overall relative risk of 3.04 (1.10–8.43) in case–control studies [7]. Additionally, recent research findings suggest that periodontitis increases the risk of IS by approximately 1.6 times [40]. Not only is IS more likely with periodontitis, but periodontitis also exacerbates the course of IS, leading to worse neurological outcomes in patients with periodontitis compared to those without it [23, 41].

In conclusion, periodontitis is an independent risk factor for the occurrence of IS, and regular dental care plays a crucial role in reducing this risk [42, 43, 44, 45]. However, results may vary based on the clinical classification of periodontitis. The aggressive periodontitis (AgP) and CP are the most common clinical type of periodontitis, the former with high incidence and rapid progression, and the latter is more prevalent among the elderly population and approximately 85% of patients may not experience disease progression within 5–6 years [9]. However, these two subtypes exhibit unequal associations with stroke outcomes. Multiple observational studies have indicated that CP patients face an elevated risk of IS when compared to individuals without periodontitis, establishing a clear causal relationship between the two conditions [46, 47, 48]. Furthermore, individuals with a history of stroke who also suffer from severe periodontitis demonstrate an increased likelihood for recurrent vascular events [49, 50]. A large cohort study with a follow‐up period exceeding 15 years found that CP is an independent risk factor for IS [51]. AgP, characterized by rapid attachment loss and alveolar bone resorption, represents a more severe form of periodontitis [52]. Comparing to CP, the association between AgP and IS may be less pronounced, possibly due to its acute and severe nature, particularly in young individuals who tend to prioritize dental care. Nevertheless, establishing a causal relationship between AgP and stroke necessitates further comprehensive and definitive investigations.

### 4.2 The Link Between Oral Microbiota and Ischemic Stroke

Compared with intestinal flora, there have been relatively few studies exploring the relationship between oral microbes and stroke (Table 1). Periodontitis is primarily caused by an imbalance of oral microbiota leading to infection. In 1989, Syrjanen et al. [54] found a link between severe oral infections and IS development in men, marking the first exploration into the connection between oral health and cerebral infarction. Subsequently, with advancements in detection technology, several studies have confirmed this association. For instance, a case–control study involving 134 elderly Chinese women with IS [55] collected pre‐diagnosis mouthwash samples for shotgun metagenomic sequencing analysis. The results showed significant differences in Alpha and Beta diversity among those who had suffered from strokes compared to controls. The subcommunities of *Corynebacterium* and *L. mirabilis* as well as *Neisseria elongata* species were significantly associated with reduced risk for IS. It was suggested that these microorganisms may interfere with stroke risk by participating in glutamic acid metabolism along with Gamma‐Aminobutyric Acid and L‐arginine pathways. In another study, higher relative abundance of *Streptococcus*, *Prevotella*, *Veillonella*, and *Fusobacterium* were observed in the oral microbiota of IS patients and high‐risk IS patients, with milder patients exhibiting better conditions compared to severe cases [56]. In clinical settings, *Streptococcus salivarius* was the most prevalent bacteria found in the mouths of stroke patients [56]. Additionally, thrombus samples contained 5.1 times more types of *Streptococcus* than arterial blood samples [57]. A study examining the oral bacterial profiles of spouses [58] revealed that stroke patients and their partners exhibited remarkably similar populations of oral bacteria. Specifically, there was a high abundance of *Fusobacterium*, *Capnocytophaga*, *Actinomyces*, *Eikenella*, and *Selenomonas* in both groups, which has been found to be associated with stroke risk factors such as diabetes and atherosclerosis. Importantly, this phenomenon also associated with an increased risk of experiencing a stroke in the partner. Further studies have directly observed an elevated abundance of *P. gingivalis* within the periodontal pockets of patients diagnosed with IS [59]. These findings suggest that certain components of the oral microbiome can be considered independent predictors for IS. For example, the measurement of serum IgG levels against periodontal pathogens can offer valuable prognostic information over a 3‐month period [60]. Notably, the presence of antibodies targeting *A. actinomycetemcomitans* and *P. gingivalis* has been linked to an increased risk of stroke [61]. Recently, emerging evidence has further suggested that the disruption of oral microbiota also contributes to the development of stroke complications and impacts clinical outcomes. Stroke‐associated pneumonia (SAP) stands out as the most prevalent complication in stroke patients [62]. There were significant dynamic variations observed in both diversity and composition of microbiota between SAP and non‐pneumonia groups, characterized by an overgrowth of aerobic bacteria, Gram‐negative bacteria, potential pathogens, biofilm‐forming bacteria, and oxidative stress‐tolerant bacteria [63]. These profound alterations occur concomitantly with disease progression. Multiple studies have demonstrated that improved oral care for stroke patients can effectively reduce the incidence of SAP, suggesting that oral microbiota holds great promise as a novel, non‐invasive diagnostic biomarker and adjunct therapeutic target in the clinical management of SAP patients.

**TABLE 1:** Alterations in the composition of oral microbiota following ischemic stroke (IS).

Subjects	Sample	Research Methods	Major changes in the oral microbiota	References
Elderly Chinese women with IS	Buccal samples	Shotgun metagenomic sequencing	Significantly different of genus Corynebacterium and Lautropia, and species *Lautropia mirabilis* and Neisseria elongate	[47]
IS, high‐risk IS (HRIS), and healthy control (HC) individuals.	Saliva	16S rRNA gene sequencing	Higher relative abundance of g_Streptococcus, g_Prevotella, g_Veillonella, g_Fusobacterium, and g_Treponema in HRIS and IS compared with that in HC	[48]
Poststroke patients and their partner	Swabs from subgingival surfaces	16S rRNA gene sequencing	Differentially abundant taxa of Bacteroidia, Prevotellaceae, Fusobacteriota, Campilobacterota, Cardiobacteriaceae, etc. in stroke group	[50]
Patients with ischemic cerebrovascular episode	Subgingival plaque samples	Conventional and real‐time PCR	Increased numbers of sites that were contaminated with *Porphyromonas gingivalis*	[52]
Patients with stroke	Saliva, swabs from the buccal mucosa, tongue, gingiva and hard pal‐ate	16S rRNA gene sequencing	A total of 103 different bacterial phylotypes	[53]

Oral microbes may exert their effects on the CNS through mechanisms which involve activation of the innate immune system, systemic inflammation leading to vascular endothelial dysfunction, enhanced cholesterol biosynthesis, and promotion of a pro‐thrombotic state [10]. These mechanisms can exacerbate atherosclerotic lesions and consequently contribute to infarctions in both large and small blood vessels in the brain. Among these mechanisms, inflammation is considered the primary mediating factor. It is well‐established that a systemic inflammatory response can further worsen local inflammation in areas of acute focal ischemia within the brain. *Porphyromonas gingivalis* , *Fusobacterium nucleatum* , and *A. actinomycetemcomitans* are pivotal pathogens in periodontitis, and the resulted elevation levels of pro‐inflammatory biomarkers such as C‐reactive protein (CRP), interleukin (IL)‐6, and IL‐1 contribute to the exacerbation of stroke by promoting neuroinflammation [64, 65]. It has been reported that patients with periodontitis and IS exhibit more severe neurological deficits [66]. Another study demonstrated that transplantation of human periodontitis saliva microbiota into the gut of mice enhanced the activation of Th17 cells and IL‐17^+^γδT cells in the small intestine, and promoted their migration from the gut to the brain [6]. This process led to rapid production of IL‐17A in brain tissue, potentially initiating an early inflammatory cascade and facilitating neuronal apoptosis, ultimately worsening prognosis. Given its pivotal role in periodontal infection, oral microflora also serves as a crucial mediator for establishing the relationship between periodontitis and IS. Further investigation is warranted regarding the impact of oral microflora on IS progression.

The precise mechanism of action of oral flora in IS apoplexy remains incompletely elucidated by current literature, and the majority of studies on this topic have focused on elucidating the association between periodontitis, oral bacteria, and conventional risk factors for stroke, which may offer valuable insights.

## 5 Periodontitis and Risk Factors for Stroke

### 5.1 Hypertension

The prevalence of both hypertension and periodontitis is high in the general population, and a causal relationship between the two exists. Individuals with periodontitis tend to exhibit hypertension while conversely, a decrease in blood pressure is associated with a reduction in periodontal pocket depth [67]. Multiple studies have reported a high detection rate of periodontitis‐associated pathogens in subgingival plaques of hypertensive individuals [68, 69, 70, 71]. Animal experiments have also confirmed that the transplantation of a high abundance of *Prevotella* from hypertensive patients into germ‐free (GF) mice resulted in an elevation in blood pressure, suggesting the potential involvement of periodontal pathogen infection in hypertension induction. Typically, bacteria are transmitted via the bloodstream and trigger an immune response that contributes to the periodontitis‐hypertension connection. The ulcer epithelium in the periodontal pocket allows bacteria and their by‐products to enter the circulation, activating the immune system and leading to increased infiltration of activated T cells such as CD4^+^T cells (especially Th1 and Th17 cells), immunosenescent (CD57^+^CD28null) CD8^+^T cells, and CD8^+^CD38^+^T cells in the blood. This process also results in elevated levels of pro‐hypertensive cytokines including IFN‐γ, IL‐17A, tumor necrosis factor (TNF)‐α, and IL‐6 [72, 73, 74]. A chronic cycle of immune activation, inflammation, and oxidative stress collaborates to drive disease progression by promoting endothelial dysfunction and vascular damage.

Oral bacteria may modulate blood pressure by influencing gut ecology. Clinical data have revealed that oral‐derived bacteria such as *Veillonella*, *Streptococcus*, *Haemophilus*, and *Prevotella* colonize the gut in hypertensive patients [75]. Among them, all species of the *Veillonella* genus are enriched in the intestines of hypertensive individuals, with a higher abundance correlating to elevated blood pressure levels, suggesting that ectopic colonization by oral *Veillonella* is a key factor contributing to the development of hypertension [76]. *Veillonella* is an important oral nitrate‐reducing bacterium which can convert dietary nitrate into NO through the NO3‐NO2‐NO pathway to maintain endothelial function and blood pressure homeostasis. Impaired bioavailability of NO resulting from *Veillonella* intestinal transport may be responsible for elevated blood pressure [68]. Senkus et al. [77] reported that eradication of oral bacteria using 0.2% chlorhexidine mouthwash twice daily significantly increased nitrite concentrations in saliva and plasma, and clinically manifested as an increase in systolic blood pressure by 2.3 mmHg. In the context of periodontitis, the oral cavity inadvertently promotes hypertension development via the oral–gut–bacterial interaction. Uncontrolled hypertension exerts a prolonged impact on blood vessel walls and damages the intima layer, consequently accelerating the development of IS.

### 5.2 Atherosclerosis

Atherosclerosis is a chronic arterial disease characterized by the formation of plaques and inflammation in blood vessels, ultimately leading to IS. Periodontitis is considered an important independent risk factor for atherosclerosis [78, 79], and the unique role of oral bacteria in the formation of atherosclerosis has been gradually discovered and appreciated. *Tannerella forsythia* , *P. gingivalis* , and *T. denticola* were frequently detected as members of the red complex in biological samples from patients with atherosclerosis [64, 80, 81, 82]. Further studies in different animal models have shown that *P. gingivalis* infection increased atherosclerotic plaque volume in mice, and initiated or exacerbated atherosclerotic lesions in normocholesterolemic/hypercholesterolemic pigs [83]. Chronic vascular inflammation, characterized by innate immune activation, plays a crucial role in the association between atherosclerosis and periodontitis. *Porphyromonas gingivalis* infection can lead to Th17/T regulatory (Treg) imbalance and further stimulate immune response [84]. Cai et al. observed a significant increase in Th17 cells and related molecules in the spleen and heart of apolipoprotein E (ApoE^−/−^) mice during *P. gingivalis* ‐induced atherosclerosis [85]. It has been reported that *P. gingivalis* inhibits the activation of Treg subsets in patients with atherosclerosis compared to non‐atherosclerotic patients and healthy controls, as well as reduces the concentration of TGF‐β1, which plays a crucial role in Tregs development [86]. Clinical studies have also revealed immuno‐colocalized of *P. gingivalis* and dendritic cells (DCs) within atherosclerotic plaques in patients with periodontitis [87]. *Porphyromonas gingivalis* invades DCs in the periodontal tissue or peripheral blood via its unique fimbrial adhesin that interacts with C‐type lectin DC‐specific ICAM3‐grabbing non‐integrin to initiate site‐specific immune responses and achieve systemic dissemination facilitated by dendritic cell's high migratory ability [88]. *Porphyromonas gingivalis* also activates the NLRP3 inflammasomes followed by macrophage pyroptosis and contributes to the formation of lipid‐rich necrotic core, which is considered to be a high‐risk vulnerable plaque [89].

Oral bacteria may disrupt gut microbial homeostasis along the oral–gut axis, thereby contributing to the development of atherosclerosis. *Porphyromonas gingivalis* , the most elucidated pathogen of periodontitis, has been found to attenuate the diet‐induced lipid‐lowering effects, which is linked to dysbiosis in the gut microbiota [90]. Oral inoculation with *P. gingivalis* for ApoE‐deficient mice reduced the diversity of gut microbiota, decreasing *Firmicutes*, increasing *Bacteroidetes*, and exacerbated atherosclerosis [91]. The composition of gut microbiota was also found to affect lipid profiles including high‐density lipoprotein, low‐density lipoprotein, and triglyceride. In another study involving ApoE^−/−^ mice [92], experimental periodontitis induced dysbiosis within the gut and led to an increase in circulating trimethylamine N‐oxide (TMAO) levels. Elevated circulating TMAO levels were also observed in patients with clinical stage III‐IV periodontitis and were associated with vascular endothelial dysfunction [93]. TMAO is a metabolite produced by gut microbiota that may increase risk for developing atherosclerosis by promoting inflammatory mediators, endothelial cell adhesion, and foam cell formation, while reducing reverse cholesterol efflux. Future treatment options aimed at addressing periodontal issues may potentially improve intestinal metabolic abnormalities, thereby alleviating or even halting the progression of atherosclerosis.

### 5.3 Diabetes Mellitus

Numerous studies have highlighted the intertwined pathogenesis between diabetes and periodontal disease [94, 95, 96, 97]. In terms of diabetes, the abnormal oral bacterial spectrum in patients with periodontitis is a significant contributor to glucose metabolism and regulation disorders [95, 96, 97]. Metabolites (LPS and gingipains) and cytokines (CRP, fibrinogen, IL‐6, IL‐1, TNF‐α) overproduced by periodontal pathogens spread into the bloodstream and interact with TLR2 and TLR4 to recruit, activate, and differentiate specific immune cells including polymorphonuclear neutrophils, mononuclear phagocytic cells, DCs, adaptive immune CD4^+^T cells, CD8^+^T cells, and B cells, resulting in a long‐term systemic low‐grade inflammation state in the host. Moreover, it can inhibit insulin receptor function and downstream signal transduction or impair the function of islet β‐cells, and leading to insulin resistance eventually [98, 99, 100]. Continuous infusion of *P. gingivalis* in mice fed a high‐fat diet not only caused endotoxemia and inflammation in liver and adipose tissue—critical sites for glucose metabolism—but also led to glucose intolerance and insulin resistance [101]. In patients with type 2 diabetes (T2DM), improvements in plasma glucose concentrations and glycated hemoglobin levels are paralleled by reductions in systemic inflammatory markers following effective periodontal treatment [102].

Periodontitis‐induced diabetes may also involve intestinal dissemination. Previous studies have demonstrated that periodontitis induces a significant reduction in alpha diversity of intestinal flora and an overgrowth of opportunistic pathogen and lipopolysaccharide‐producing bacteria such as *Deltaproteobacteria*, *Escherichia‐Shigella*, and *Enterobacteriaceae*; with a decrease in butyric acid‐producing bacteria [103, 104]. These changes align with the characteristics observed in the intestinal microbiota of diabetic animals/patients and may negatively impact blood glucose homeostasis [105]. In obese mice mimicking T2DM, treatment with *P. gingivalis* resulted in increased fasting plasma glucose levels while disrupting the gut microbiome at the phylum, family, and genus levels [106]. This disruption facilitated the transportation of low molecular weight bacterial metabolites to the liver, impairing glucose tolerance and enhancing insulin resistance. Oral administration of *P. gingivalis* led to an increase in the proportion of *Bacteroidetes* within the ileum, accompanied by insulin resistance along with upregulation of pro‐inflammatory genes in adipose tissue and the liver, while downregulating anti‐inflammatory or insulin sensitivity‐improving genes which elevated serum glucose levels and caused insulin intolerance [107]. The elevation of serum endotoxin induced by periodontitis is closely correlated with the level of HbA1c. However, the elimination of ligation or cohabitation with healthy mice to restore intestinal flora, as well as antibiotic treatment for gut microbiota eradication, significantly diminishes the levels of serum endotoxin in mice with periodontitis [103]. This implies that a fraction of circulating endotoxin in mice may originate from the gastrointestinal tract. *Porphyromonas gingivalis* ‐mediated dysbiosis of gut microbiota also exacerbates clinical symptoms of type 1 diabetes. In streptozotocin‐induced diabetic mice, a decrease in *Lactobacillus* and Turicibacter populations and an increase in *Staphylococcus* were observed within intestinal tissues [108]. *Lactobacillu*s is known to enhance barrier integrity [109], while butyric acid produced by *Turicibacter* contributes to insulin sensitivity [110], and *Staphylococcus* is associated with pro‐inflammatory responses [108], which are aligned with higher fasting plasma glucose concentrations and inflammatory gene expression levels. These findings support the hypothesis that oral bacteria colonize along the gastrointestinal tract and subsequently influence gut microbiota composition and metabolism toward a pro‐diabetic state, thereby increasing stroke risk with the oral–gut axis acting as a pivotal link.

### 5.4 Obesity

Obesity is defined as the abnormal or excessive accumulation of adipose tissue [111]. Periodontitis is now recognized to exacerbate obesity. Numerous epidemiological investigations and animal studies have consistently reported a positive correlation between periodontitis and obesity, establishing a bidirectional relationship [112, 113, 114, 115]. The association between obesity and periodontitis shares similarities with the association of periodontitis and diabetes, specifically involving alterations in the microenvironment of oral flora [116, 117, 118, 119, 120, 121], immune factors and inflammatory responses, and the transportation of pathogenic bacteria from oral lesions to the intestine. Elevated levels of TNF‐α, IL‐6, and CRP in the plasma of periodontitis patients are closely associated with obesity [122]. A transcriptomics study of adipose tissue in obese patients and periodontal tissue in periodontitis patients indicated that the characteristic genes shared by the two groups were closely linked to immune cell function, particularly macrophage activation, migration, and infiltration [123].

Periodontitis is also linked to obesity through intestinal microbiota alterations. Dong et al. observed a decrease in the *Firmicutes*/*Bacteroidetes* (F/B) ratio in both obese patients and *P. gingivalis* ‐treated mice, accompanied by adipocyte hypertrophy, insulin resistance, adipose tissue inflammation, and intestinal barrier defects—similar to high‐fat diet‐induced obese mice [124]. Alterations in specific by‐products of the gut microbiota provide insight into the mechanisms underlying the contribution of the oral microbiota to obesity. Circulating concentrations of TMAO were found to be positively correlated with body mass index in a dose‐dependent manner. However, under the influence of *P. gingivalis* , there was an increase in the proportion of *Prevotella* within the gut microbiota and enhanced choline metabolism leading to elevated levels of TMA/TMAO [125]. Furthermore, oral administration of *P. gingivalis* significantly augmented aromatic amino acid production compared to sham‐administered mice, which has been reported to be associated with risk of obesity previously [126]. Although the available data are limited, these findings provide sufficient evidence to suggest that the “oral–gut axis” may serve as a plausible explanation for the impact of periodontitis on the development of obesity. Periodontal pathogens are ingested or transported into the gastrointestinal tract through the bloodstream, leading to disruption in both structure and function of the gut microbial community in situ. This disruption acts as a catalyst for the development of obesity and establishes a positive feedback loop between these two diseases.

Hypertension, atherosclerosis, diabetes, and obesity are significant modifiable risk factors for IS. Following the onset of periodontitis, associated pathogens trigger the host immune response, or migrate to the gut and establish novel interactions with resident bacteria, thereby contributing to the pathophysiological mechanisms underlying these diseases. The gut microbiota is widely recognized as the central component of the brain–gut axis, and its mediated disruption in intestinal ecology can elicit changes in the central nervous system (CNS), while oral pathogen‐mediated inflammation has also been implicated as a contributor to the development of cerebrovascular disease. There appears to be interplay between the oral cavity, gastrointestinal tract, and brain.

## 6 Oral–Gut–Brain Axis

### 6.1 Oral–Gut Microbial Transmission

The oral cavity and gastrointestinal tract are the two largest microbial ecosystems within the human body. Recent evidence challenges the conventional notion that transmission of oral microbiota to the gut is hindered by physiological segregation, suggesting that transfer can occur under conditions of immature or impaired oral–gut barrier function [127]. Stool samples from patients treated with proton pump inhibitors (PPIs) exhibit an overrepresentation of oral bacteria such as *Rothia*, *Scardovia*, *Actinomyces*, and *Micrococcaceae* [128]. Similarly, oral bacteria including *Porphyromonas*, *Fusobacterium*, *Eggerthella*, *Corynebacterium*, and *Pseudoramibacter* are commonly found in the gut of elderly individuals [129]. In periodontitis cases, various pathogens like *P. gingivalis* , *F. nucleatum* , *Streptococcus mitis* /*parasanguinis*, and *Parvimonas micra* can be transmitted to the intestine through saliva [130]. In contrast, direct transfer of intestinal bacteria to the oral cavity is infrequent with fecal‐oral transmission probably being the primary route for bacterial dissemination. A study examining microbe samples from stool, mouth, and hand revealed that 48.9% of participants had detectable fecal bacterial signals on their palms while 67.2% exhibited oral signals on their palms [131]. Even among healthy individuals, colonization from gut to mouth occurs. *Bifidobacterium* represents a predominant genus in newborns' intestinal tracts but can also be detected in saliva [132]. One study demonstrated that 89% of duodenal bacteria were present in matched oral samples [133]. Analogously, analysis of salivary and fecal microbiota from 470 individuals (310 samples) revealed that both sample types shared 125 species, including *Streptococcus*, *Veillonella*, *Actinomyces*, and *Haemophilus* [127].

Currently, it is widely accepted that the oral microbiome is transmitted to the gut through two primary mechanisms. Direct invasion occurs as oral microbes enter the intestinal tract via the esophagus during swallowing. An average individual ingests approximately 1.5 L of saliva and millions of microorganisms daily [134]. However, under physiological conditions, most of these microorganisms perish upon crossing the gastrointestinal barrier due to stomach and bile acids [12]. Following a pathological insult, when the oral pathogen load surpasses the threshold of resistance of digestive barriers, oral microorganisms can colonize the gut and become opportunistic pathogens. Reduced stomach acid induced by Long‐term use of PPIs damages the effective antibacterial barrier between mouth and gut thereby facilitating translocation of oral microbiota into the gut environment [135]. The blood‐borne pathway may indirectly serve as a transmission route for oral microorganisms [136]. Oral bacteria have demonstrated an ability to invade immune cells such as DCs and macrophages while utilizing these immune cells as delivery vectors for transfer to the intestinal mucosa [137]. Recent studies have confirmed that the CP‐induced proliferation and dilation of periodontal blood vessels facilitates the infiltration and dissemination of microorganisms beyond the oral cavity, into areas such as the joints and colon [138]. During transient physiological bacteremia in the mouth, *Fusobacterium* appears to exploit the circulation system as a highly effective route for reaching colorectal cancer, with evidence of its presence also found in liver metastases [136, 139].

Oral microorganisms entering the gastrointestinal tract can induce alterations in the structure of the intestinal microbial community to a certain extent. A decrease in the F/B ratio is the main impact of oral gavage with *P. gingivalis* or *A. actinomycetemcomitans* [140]. *Clostridium butyricum* , a member of Firmicutes, produces butyric acid through anaerobic fermentation, which suppresses IL‐17 secretion while eliciting Treg responses [141]. Conversely, *Bacteroidetes* increase intestinal SCFAs levels, resulting in enhanced expression of pro‐inflammatory response‐related genes, heightened reactivity toward Th1 and Th17 cells of the intestinal immune response [142]. Other studies [39] have demonstrated that induction of experimental periodontitis with *P. gingivalis* impairs intestinal barrier function by downregulating tight junction protein 1, claudin‐1, and occludin expression in intestinal epithelial cells, which hinders proper establishment of tight junctions and increases intestinal permeability, facilitating invasion of pathogenic factors into the bloodstream. Consequently, metabolic endotoxin production occurs and further induces inflammation‐related changes in various tissues and organs. Inflammatory bowel disease (IBD) serves as a typical example illustrating this phenomenon. Clinical studies have consistently demonstrated that oral lesions are frequently observed in patients with IBD, often appearing as an initial symptom and closely associated with disease activity [143]. Disruption of intestinal mucosal homeostasis allows oral flora to invade through ulcerated epithelium within periodontal pockets and colonize the intestine. Notably, patients with IBD exhibit a notable enrichment of *Porphyromonas*, *Prevotella*, and *Gemella* oral bacteria within their intestinal mucosa [144]. Furthermore, research has indicated that *P. gingivalis* utilization can lead to dysregulation of intestinal flora and impaired barrier function while also upregulating various pro‐inflammatory cytokines [138]. The experimental animal oral–gut axis may link periodontitis and IBD through immune mechanisms [145]. *Enterobacteriaceae* in the oral cavity but not isolated from the gut are known to induce intestinal inflammation by activating inflammatory vesicles in macrophages, mediating IL‐1 signaling [146]. *Fusobacterium nucleatum* also increases intestinal inflammation by promoting the release of pro‐inflammatory cytokines TNF‐α, IFN‐γ, IL‐1β, IL‐6, and IL‐17 but decreasing the release of anti‐inflammatory cytokine IL‐10 [147]. The dysregulation of T‐cell immunity and its secretory mediators is key to the pathogenesis of IBD. *Porphyromonas gingivalis* indirectly induces an increase in the percentage of IL‐9^+^CD4^+^T cells in small intestinal lamina propria lymphocytes and downstream inflammation by altering intestinal flora [148]. Oral pathogen‐specific Th17 cells, notably from *Klebsiella* spp. and *Enterobacter* spp., express gut‐homing molecules CCR9 and α4β7 on their cell surface. These cells possess the ability to migrate from the oral mucosa to the gut and undergo a phenotypic transformation from Th17 (RORγt^+^ T‐bet^−^) to a mixed Th1/Th17 phenotype (RORγt^+^ T‐bet^+^) that produces both IL‐17A and interferon‐γ, which aligns with findings in the intestinal mucosa of IBD patients [149]. The balance between Th17/Treg is intricately related to bidirectional interaction between periodontitis and IBD. *Porphyromonas gingivalis* aggravates intestinal inflammation by upregulating expression of TH17‐related transcription factors and production of pro‐inflammatory factors such as IL‐17 and IL‐6 while downregulating expression of Treg transcription factor Foxp3 and production of anti‐inflammatory factors like TGF‐β and IL‐10 through the TLR4 pathway, while fecal microbiota transplantation has shown efficacy in treating both intestinal and oral inflammation [150]. These findings provide compelling evidence supporting the existence of an “oral–gut” axis wherein microbial aggregation commences from the oral cavity toward its culmination in the intestine and interspersed with multiple immune signals—a symbiotic relationship crucial for regulating physiological functions and pathological processes.

### 6.2 Gut–Brain–Microbiome Axis

The brain and the gut maintain a continuous and extensive bidirectional communication, encompassing multiple feedback loops including the CNS, endocrine signaling pathways, immune regulation, and metabolic effects [151]. The gut‐residing microbiota plays a pivotal role in facilitating gut–brain communication, with most signals derived from the microbiota being directly transmitted from the enteric nervous system to the brain via vagal afferent fibers within the neural network; subsequently triggering neuromodulation, neurotransmitter release, and immune signal‐mediated reflex responses [151, 152]. Indirectly, signals such as immune cells and factors [153], bacterial metabolites [154], neurotransmitters [155], and intestinal cell secretions [156] are conveyed to regulate brain structure, function, and development through systemic circulation. Simultaneously, they reciprocally influence gastrointestinal motility, immune response, and secretory functions, as well as composition/metabolism of the microbiome—thus completing bidirectional communication along the brain–gut axis. Furthermore, the hypothalamic–pituitary–adrenal (HPA) axis plays a crucial and dynamic role within the microbiome–gut–brain axis [157]. Studies conducted on GF rodents have demonstrated that excessive activation of the HPA axis disrupts circulating levels of pro‐inflammatory cytokines, such as IL‐1β, IL‐6, and TNF‐α, thereby compromising the integrity and functionality of the intestinal microbiota and leading to detrimental outcomes through the gut–brain axis [158]. Elevated concentrations of corticotropin releasing hormone also contribute to increased intestinal permeability which facilitates microbial translocation across epithelial barriers [159].

Currently, the neuroimmune communication within the gut–brain axis has been extensively investigated. Numerous studies have demonstrated that alterations in intestinal flora composition and metabolite levels impact the overall immune status of the host and are associated with the onset, progression, and prognosis of various neurological disorders. Mesenteric lymph nodes harbor 70%–80% of inflammatory and immune cells in the body, and disruption of both the blood–brain barrier and intestinal epithelial barrier under pathological conditions will trigger migration of a large number of these cells to the brain [160]. LPS, a significant product of Gram‐negative microorganisms, binds to TLR to activate immune cells such as DCs, neutrophils, and macrophages, subsequently leading to production of pro‐inflammatory cytokines including IL‐1α, IL‐1β, TNF‐α, and IL‐6 which traverse the blood–brain barrier impacting neurological function [53]. Furthermore, gut microbial signaling appears to profoundly influence brain resident immune cells particularly microglia development, maturity, and astrocyte activation [161]. CNS diseases can also alter intestinal flora composition stimulating loss of intestinal immune homeostasis. IS [162] and traumatic brain injury [53] promote proliferation of Th1 and Th17 cells, inhibit differentiation of Treg cells, and stimulate exacerbation of intestinal inflammation. In summary, the innate immune response is mediated by innate immune cells such as neutrophils, microglia, macrophages, mast cells, γδTregs, and NK cells, while adaptive immunity is primarily mediated by T lymphocytes and B lymphocytes, and these immune cells together with their secreted chemokines and cytokines actively participate in the inflammatory and immune responses associated with gut–brain axis‐related diseases, serving as a major regulator of gut–brain interactions.

### 6.3 Oral–Microbiota–Brain Crosstalk

Disruption of the oral microbiota is increasingly being associated with nervous system‐related disease through a communication pathway known as the oral microbiota–brain axis. Studies in animal models and postmortem brain tissue from Alzheimer's disease (AD) patients strongly suggest that *P. gingivalis* and/or its product, gingipain, translocate to the brain and lead to β‐amyloid plaque formation, which subsequently triggers neuronal death [163, 164, 165]. Kamer et al. [166] found an increased detection rate of periodontal bacterial antibodies in AD patients, which were independently correlated with AD. In mice, collagen‐binding activity exhibited by Cnm‐positive *S. mutans* enables it to exit the oral niche and enter the brain to cause or exacerbate intracerebral hemorrhage [167]. Central factors also influence the composition of oral cavity microbiota. Numerous preclinical and clinical studies [1, 168, 169] have implicated psychological stress in altering taxonomic abundance of *Prevotella*, *Neisseria*, *Haemophilus*, *Spirochaetes*, *Corynebacterium*, and *Fusobacterium*. Stress‐related hormones including epinephrine, norepinephrine, and cortisol along with AI‐though mucin affect *F. nucleatum* , *P. gingivalis* , and *Actinomyces* viability. The crosstalk between the mouth and the brain is also bidirectional but exhibits a slightly distinct pathway of action compared to that observed in the gastrointestinal tract, likely attributed to their anatomical proximity.

The trigeminal/olfactory/facial nervous system serves as a direct route for oral bacteria and their products to affect the brain. Notably, *T. denticola* , an oral bacterium, has been detected in the trigeminal ganglion and hippocampus of AD individuals, and it has been found to induce hippocampal amyloid‐β production in mice [170, 171]. Riviere et al. [167] detected *Treponema* in human frozen samples of trigeminal ganglia, brain stem, and cortex. Transient bacteremia resulting from activities such as tooth brushing, tooth extraction, or oral inflammation allows pathogenic microorganisms to breach the oral mucosal barrier from sources like root canals or gingival connective tissue capillaries into the bloodstream [142]. Although these microorganisms typically do not persist in circulation for long periods of time, under conditions where blood–brain barrier permeability is increased they are able to penetrate it, carrying toxic factors into brain tissue where they proliferate and cause disease development, similar to *P. gingivalis* [142]. Moreover, certain oral bacteria along with their secreted cytokines and toxic substances may alter the permeability of the blood–brain barrier, causing damage to the CNS. Previous studies have reported that mice infected with *Streptococcus* gingivalis exhibited compromised integrity of their blood–brain barriers as well as reduced levels of tight junction‐related proteins compared to uninfected controls, wherein the outer membrane vesicles (OMVs) secreted by *P. gingivalis* play a crucial bridge role. Due to their smaller size compared to bacteria, OMVs enable *P. gingivalis* to efficiently cross the blood–brain barrier by degrading endothelial adhesion protein PECAM‐1, β‐1 integrin, and extracellular matrix (ECM) proteins. Alternatively, they can be phagocytosed by macrophages and transported to the brain where they release virulence factors that activate neuroinflammation, neurofibrillary tangle formation, ferroptosis, and other processes associated with accelerated AD‐specific neuropathological responses [172]. Like gut microbiota, the oral microbiota also interacts with the host through the HPA axis. Elevated cortisol levels result in upregulation of virulence factors from specific microorganisms like Fusobacterium and *P. gingivalis* , which is associated with increased susceptibility to oral inflammation [173]. Consequently, a heightened bacterial load exacerbates psychiatric disorders such as bipolar affective disorder, which also impacts catecholamine levels [174].

Pathogenic microorganisms colonized in brain tissue contribute to neuroinflammation. *Porphyromonas gingivalis* modulates the host immune response by disrupting complement factors for bacterial resistance promotion. Periodontitis induces systemic inflammation, and increased pro‐inflammatory cytokines activate the endothelial cells to express TNF‐α and IL‐1 receptors, and drive perivascular macrophages adjacent to brain endothelial cells to communicate with microglia, leading to microglia activation and neuroinflammation [175]. In the rat brain, it was observed that LPS derived from *P. gingivalis* triggered the polarization of M1 microglia/macrophages through the TLR4/NK‐κB signaling pathway, increasing the expression of CD86 and iNOS markers as well as related inflammatory factors TNF‐α, IL‐1β, and IL‐6 [176]. *Spirochete* is believed to activate TLRs on glial cells through CD14, thereby stimulating production of pro‐inflammatory cytokines [177]. In addition, modulation of microbial community composition may impact brain function by influencing specific neural pathways. The oral microbiome can directly impact the brain's reward circuitry associated with smoking behavior and dependence through neurotransmitter‐related pathways [178]. A disruption in the *Prevotella* co‐occurrence network has also been observed in autism spectrum disorder patients' plaques, potentially contributing to cognitive and behavioral changes [179].

### 6.4 The Oral–Gut–Brain Axis

The oral microbiota has the potential to migrate to the gut and modulate the intestinal microecosystem, while the gut interacts with the brain. No study has yet investigated possible communication between the mouth and CNS through the gut pathway in organisms. Existing literature has suggested the presence of an oral–intestinal‐brain axis. *Porphyromonas gingivalis* in the gut elevates IL‐17 levels in both gut tissue and the systemic circulation, including serum and liver tissue [180, 181]. Given that IL‐17 can traverse the blood–brain barrier and stimulate inflammatory mediators, chemokines, antimicrobial peptides, as well as induce degeneration and death of dopaminergic neurons via IL‐17RA activation [181]; this regulation may operate independently from both oral–brain axis and gut–brain axis mechanisms. However, future studies should simultaneously examine bacteria along with their associated factors at a gastrointestinal level using experimental periodontitis models to confirm or exclude any potential effects on the gut.

Based on the previously mentioned findings combined with previously collected data, we present a comprehensive analysis of an organ action network. The oral–gut–brain axis comprises four components: (1) An oral–gut axis involving exchange and functional interaction of microbiota within the digestive tract or bloodstream; (2) a gut–brain axis encompassing neural, immune, and endocrine mechanisms facilitating interactions between gut and brain; (3) an oral–brain axis mediated primarily by the trigeminal nerve transmitting microbial signals to enable communication between the oral cavity and CNS; (4) within this complex interplay of interactions in the oral–gut–brain axis, an imbalance in oral microbiota can trigger alterations in intestinal microbiota. Oral bacteria and their metabolites persisting within the gut have potential access to enteric circuits and the systemic circulation, leading to central lesions within the brain [182]. Therefore, the oral and gut microbiotas are promising therapeutic targets for diseases of the CNS such as IS.

## 7 Oral Microbiota as Therapeutic Target

### 7.1 Oral Hygiene

Oral hygiene serves as the foundation of dental health, promoting oral function and mitigating disease burden by controlling pathogenicity. Poststroke patients often harbor a higher prevalence of aerobic and facultative anaerobic Gram‐negative bacteria in their mouths, increasing the risk of transfer to the lungs and development of SAP due to difficulty swallowing. Studies have demonstrated that enhanced oral care can reduce incidence rates of SAP among stroke patients [62]. Furthermore, a comparative survey on poststroke oral care regimens revealed a 4% decrease in *Staphylococcus aureus* colonization rates with standard care compared to usual care [183]. A personalized oral care plan encompassing brushing, oral mucosa protection, and moisturizing helps maintain normal balanced flora despite cognitive/motor disorders or hospitalization‐induced neglect. While existing literature strongly advocates for professional assessment and intervention for poststroke oral health management, further research is necessary to determine optimal protocols.

### 7.2 Non‐Surgical Treatment for Periodontitis

The efficacy of mild and moderate periodontitis is primarily maintained through non‐surgical treatment. Mechanical removal of dental plaque is the most widely utilized and effective method for treating periodontal disease, including supragingival scaling, subgingival scaling, and scaling and root planing (SRP). Supragingival scaling can be categorized into two types: manual scaling and ultrasonic scaling. Manual scaling involves using a manual scaler to remove plaque and stubborn calculus from the periodontal pocket, resulting in a smooth root surface. However, it may inadvertently remove excessive cemento‐bone, making it time‐consuming and labor‐intensive. On the other hand, ultrasonic instruments not only minimize cementum removal and soft tissue trauma but also reduce reliance on operator skill while significantly reducing treatment time, which possess higher efficiency in scaling [184, 185]. Subgingival scaling refers to the utilization of delicate subgingival scaling instruments to eradicate diseased tissue and scattered dental plaque located on the root surface within the periodontal pocket, aiming to achieve a smooth root surface, which facilitates peridontal reattachment and elimination of periodontal pockets. Notably, bacteria not only reside in the periodontal pocket but also colonize other intraoral spaces like mucosa, tongue, tonsils, and saliva, which makes thoroughly remove gum biofilm under challenging, therefore supporting using antimicrobials are generally considered part of the treatment plans. Antibiotics are commonly used for systemic therapy, with the most frequently employed treatment being the combination of amoxicillin (AMX) and metronidazole (MET) [186]. Numerous studies have demonstrated that adjunctive systemic AMX + MET following SRP significantly reduces periodontal pathogens and inflammatory cytokines compared to SRP alone, as well as leading to improved clinical outcomes such as decreased probing pocket depth and increased clinical attachment level [187, 188, 189, 190]. Immunomodulatory drugs are also believed to help limit the progression of periodontitis. Non‐steroidal anti‐inflammatory drugs (NSAIDs) can inhibit cyclooxygenase‐L activity by blocking the metabolism of arachidonic acid into prostaglandins and thus prevent alveolar bone resorption [191]. However, there is still a lack of large‐scale controlled clinical studies on the use of NSAIDs in the treatment of periodontal diseases, and the potential adverse effects requires further observation and evaluation. Studies have also indicated that the complement system plays a role in the occurrence and development of periodontitis [192, 193, 194, 195]. Certain complement‐specific drugs like C5a receptor inhibitor PMX‐53 can effectively inhibit overgrowth of *P. gingivalis* and other oral microorganisms with good safety profiles [196], which hold promise as adjuvant therapies for periodontitis.

### 7.3 Surgical Treatment for Periodontitis

As periodontitis progresses to an advanced stage and non‐surgical treatment proves ineffective, surgical intervention becomes necessary. The primary objective of periodontal surgery is to eliminate diseased tissue within the periodontal pocket, stimulate regeneration of the periodontal tissues, correct anatomical irregularities, prevent plaque accumulation and recurrence of pocket formation, as well as address aesthetic concerns arising from contour defects. Currently, surgical treatments for periodontal disease primarily encompasses resection, reconstruction, and regeneration techniques. Resective surgery removes necrotic or diseased alveolar bone or gingival tissue to reconstruct its normal physiological morphology [197]. Regenerative surgery focuses on repairing alveolar bone using synthetic graft materials or autogenous bone grafts to establish new periodontal attachments and enhance tooth support [198]. In recent years, in order to enhance the efficacy of periodontal regeneration, biological modifications have been employed in conjunction with bone replacement and barrier membranes. Purified recombinant human platelet‐derived growth factor BB (rhPDGF‐BB) represents a multipotent cell growth factor capable of stimulating proliferation and recruitment of both periodontal ligament cells and osteocytes [199]. A meta‐analysis revealed that the addition of rhPDGF‐BB during bone grafting exhibited a superior capacity for promoting periodontal tissue regeneration [200]. Similarly, enamel matrix derivatives (EMD) also demonstrated favorable outcomes in repairing periodontal defects, with EMD‐treated inferior bone defect sites exhibiting a greater degree of enhanced clinical attachment [201]. Furthermore, with advancements in tissue engineering technology and stem cell therapy, the utilization of stem cell transplantation for tooth regeneration and periodontal repair has emerged as an area of significant interest. However, most current research remains confined to laboratory investigations, posing substantial difficulties and challenges for clinical translation.

### 7.4 Prebiotics and Probiotics

Prebiotics and probiotics are first‐line therapeutic interventions for modulating the composition of the microbiota. Probiotics, as living microorganisms, can confer health benefits to the host when administered in adequate quantities [183]. Prebiotics are substrates that are selectively utilized by host microorganisms and also offer health benefits [183]. These substances play a role in shaping the microbial ecosystem of the gut and mouth through various mechanisms such as producing antimicrobial compounds, inhibiting pathogenic bacteria, modulating immune responses, and performing metabolic functions. Several clinical trials observed a reduction in periodontal pathogens during probiotic supplement treatment, as well as the concentration of IL‐17, IL‐1β, and TNF‐α in the gingival crevice fluid. Probiotic strains may also have transient colonization in the mouth [13, 202]. Oral nitrate‐reducing bacteria, represented by *Neisseria*, *Rothia*, *Actinomyces*, and *Kingella*, possess the capability to convert salivary nitrate into nitrite, which prevents oral diseases and increase systemic nitric oxide levels, thereby improving hypertension and diabetes [203]. Unlike probiotics, prebiotics have only recently emerged in the field of oral health with limited research conducted thus far. The frequent metabolism of sugars by the oral microbiota leads to acidification of the ecosystem. Urea and arginine are the main sources of alkaloids in the oral cavity, which have been suggested as potential prebiotics that can limit the development of dental caries [204]. Nitrates represent another promising prebiotic candidate, and a clinical study showed that consuming nitrate‐rich lettuce juice for 2 weeks reduced gum inflammation [203]. Treatment with N‐acetyl‐D‐mannosamine, succinic acid, and Met‐Pro di‐peptide resulted in a significant increase in the proportion of beneficial bacteria species and a decrease in the proportion of pathogenic bacteria species within multi‐species biofilms, indicating a potential prebiotic effect [205]. However, there are still unresolved issues pertaining to the modification of these microbiomes. When selecting appropriate probiotics or prebiotics, it is imperative to assess the microbiome interactions and consider dynamically changing environmental factors that may influence therapeutic outcomes.

### 7.5 Nanoparticle Drug Delivery System

The hydrogel platform presents a promising and distinct strategy for the treatment of periodontitis. It offers ease in preparation and administration, enables filling irregular periodontal defects, and effectively maintains the biological activity of drugs while controlling their release kinetics [206]. To date, hydrogels have demonstrated the potential to alleviate periodontitis through mechanisms such as promoting bone regeneration, modulating immune responses, and inhibiting anaerobic bacteria [207, 208, 209]. Recently, researchers have proposed a novel composite hydrogel‐nanoparticle hybrid platform that exhibits excellent antibacterial efficacy against common oral pathogens *A. actinomycetemcomitans* , *P. gingivalis* , and *F. nucleatum* , while also aiding in restoring immune homeostasis and exhibiting therapeutic effects on both periodontitis and hypertension [206]. A silver nanoparticle (AgNP)‐based hydrogel has been reported to sustainably adhere to the oral cavity in chemically induced oral squamous cell carcinoma mice models, enhancing anti‐tumor immune responses by modulating the oral microbiota.

### 7.6 Biofilm Disruption

Pathogenic plaque biofilm is a structured microbial community enveloped in exopolysaccharides (EPS) that confers a protective sheath against host defense and antimicrobial agents for pathogens [210]. The control of pathogenic biofilm holds significant clinical implications for oral and systemic health. Conventional treatment of dental plaque biofilm primarily relies on mechanical removal and antibiotics; however, the former is not entirely eradicating, while the latter may induce multidrug resistance and disturb the normal flora. The emergence of nanotechnology has introduced novel prospects for biofilm management. Zinc oxide and silver nanoparticles, as well as iron oxide nanomaterials, have been proposed as antibiotic coating agents [211]. Notably, glucan‐coated iron oxide nanomaterials can selectively target biofilm cells within a pathologically acidic environment and facilitate EPS matrix degradation through H_2_O_2_ mediation, thereby impeding oral biofilm‐associated infections [211]. Ultrafine gold nanoclusters (AuNCs) exhibit exceptional permeability properties that significantly inhibit *F. nucleatum* , inducing in vivo biofilms while dismantling mature in vitro counterparts, thus mitigating periodontal inflammation and bone loss [212]. CDots represent an emerging class of carbon‐based nanoparticles with studies demonstrating that function‐adaptive clustered nanoparticles can eliminate biofilm EPS content while reducing *S. mutans* viability without compromising oral ecological balance [213]. Exogenous arginine also serves as an ideal potential therapeutic agent by diminishing microbial coaggregation, modifying EPS biochemical composition, and maintaining plaque homeostasis to counteract the development of oral diseases [210]. Probiotics also exert influence on biofilm formation. One in vitro study [214] reported *Lactobacillus reuteri* strains' ability to co‐aggregate with *Candida albicans* during the process of forming a biofilm, creating an unfavorable environment that inhibits yeast growth.

## 8 Conclusion

**FIGURE 1:**
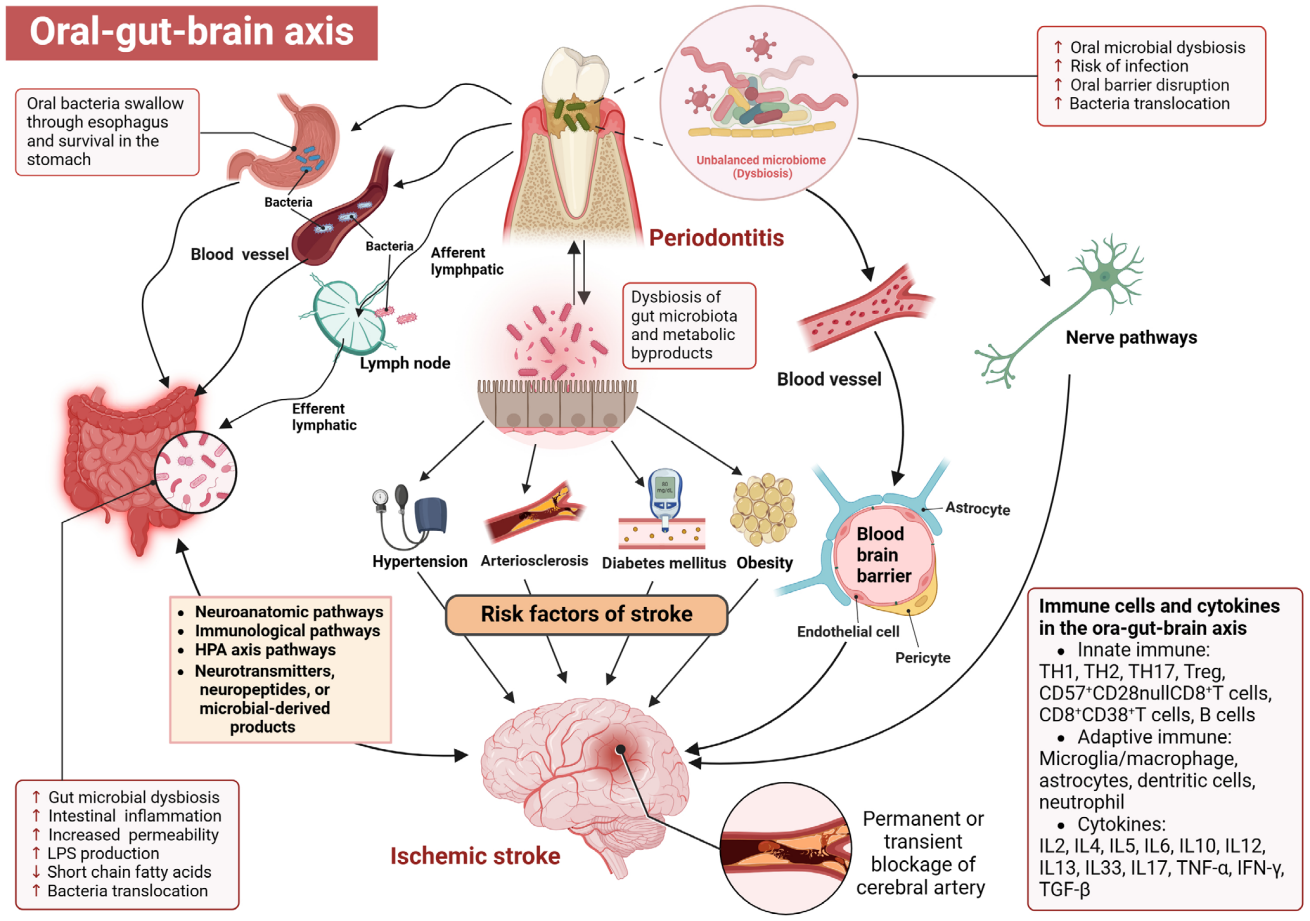
Schematic representation of the bidirectional role of the oral–gut–brain axis in ischemic stroke (IS) and periodontitis. Disruption of the oral microbial community leads to periodontitis, influencing the occurrence and progression of IS. Directly, oral bacteria and their by‐products impact the brain through both the nervous system, circulating blood and immune responses. Indirectly, pathogenic oral microorganisms modulate stroke risk by perturbing gut ecosystem/immune homeostasis via the gut–brain–microbiome axis or interacting with stroke risk factors.

The interorgan microbial network is emerging as a crucial regulator of physiological functions and pathological processes. In this review, we primarily focused on periodontitis and IS, two diseases affecting different organs in the body, aiming to establish the oral microbial–gut–brain axis in IS by analyzing their correlation (Figure 1). The association between the oral microbiome and stroke can be either direct, where oral bacterial species and their products directly impact the brain through the nervous system, circulating blood, innate and adaptive immune response, or indirect, where oral pathogenic microorganisms disrupt intestinal microbiome balance leading to changes in intestinal permeability and immune system stability. As well, interactions between oral bacteria/periodontitis and stroke risk factors may influence the risk of stroke. However, previous studies have solely examined the effects of *P. gingivalis* , thereby necessitating further comprehensive studies to elucidate the intricate relationship and detailed mechanisms underlying the oral microbial–gut–brain axis in IS within a periodontitis context. Interventions targeting oral microbiota such as effective management of oral hygiene practices, utilization of prebiotics/probiotics, nanomedicine delivery systems, and biofilm disruptors have demonstrated health benefits highlighting their potential for restoring ecological balance within the oral flora for treating periodontitis or IS. Rigorous design and implementation of basic as well as clinical studies are required to facilitate translation of research findings into clinical practice.

## Author Contributions

Z.Y. and K.X.H. jointly wrote the original draft and drew the table and figure. P.B. contributed the conception and design. B.X.F. and S.D. contributed to modify and polish the language. Z.L. reviewed and edited the manuscript. X.X.X. supervised the work and prepared the final version. All authors read and approved the final manuscript.

## Ethics Statement

The authors have nothing to report.

## Conflicts of Interest

The authors declare no conflicts of interest.

## Data Availability

The authors have nothing to report.
